# Physical exercise mediates a cortical FMRP–mTOR pathway to improve resilience against chronic stress in adolescent mice

**DOI:** 10.1038/s41398-023-02311-x

**Published:** 2023-01-19

**Authors:** Lan Yan, Mei Wang, Fengzhen Yang, Yajie Wang, Siqi Wang, Kwok-Fai So, Li Zhang

**Affiliations:** 1 Key Laboratory of CNS Regeneration (Ministry of Education), Guangdong-Hong Kong-Macau Institute of CNS Regeneration, Jinan University, Guangzhou, China; 2 College of Life Science and Technology, Jinan University, Guangzhou, China; 3 State Key Laboratory of Brain and Cognitive Science, Li Ka Shing Faculty of Medicine, The University of Hong Kong, Hong Kong SAR, China; 4 Center for Brain Science and Brain-Inspired Intelligence, Guangdong-Hong Kong-Macao Greater Bay Area, Guangzhou, China; 5 Neuroscience and Neurorehabilitation Institute, University of Health and Rehabilitation Sciences, Qingdao, China

**Keywords:** Depression, Molecular neuroscience

## Abstract

Aerobic exercise effectively relieves anxiety disorders via modulating neurogenesis and neural activity. The molecular mechanism of exercise-mediated anxiolysis, however, remains incomplete. On a chronic restrain stress (CRS) model in adolescent mice, we showed that 14-day treadmill exercise profoundly maintained normal neural activity and axonal myelination in the medial prefrontal cortex (mPFC), in association with the prevention of anxiety-like behaviors. Further interrogation of molecular mechanisms revealed the activation of the mechanistic target of the rapamycin (mTOR) pathway within mPFC under exercise training. At the upstream of mTOR, exercise-mediated brain RNA methylation inhibited the expression of Fragile X mental retardation protein (FMRP) to activate the mTOR pathway. In summary, treadmill exercise modulates an FMRP–mTOR pathway to maintain cortical neural activity and axonal myelination, contributing to improved stress resilience. These results extended our understanding of the molecular substrate of exercise-mediated anxiolytic effect during adolescent period.

## Introduction

Physical exercise is one effective strategy for preventing or relieving anxiety disorders. In humans, a meta-analysis showed that exercise is effective in improving anxiety symptoms in people suffering from stress-related disorders [1]. Using different animal models, physical exercise relieves anxiety-like symptoms [2–4]. In addition to these anxiety behaviors, exercise training also helped to improve the cognitive deficits induced by chronic stress [5, 6]. Neurobiological studies have proposed distinct mechanisms, including enhanced hippocampal neurogenesis [7], anti-inflammatory cytokines [8], and cerebrovascular modulations [9] under exercise training. At the cellular level, endurance exercise profoundly affects the morphology and function of neurons, astrocytes [10, 11], microglial cells [12], and oligodendrocytes [13]. Although the cellular changes under the exercise paradigm have been characterized in detail, underlying molecular mechanisms remain at an incomplete stage. Current knowledge agrees that physical exercise may potentiate hormonal factors such as brain-derived neurotrophic factor (BDNF) to counteract with anxiety or depression disorders [14]. However, the complete cellular signaling pathway of exercise-mediated anxiolysis has not been resolved.

Our group previously demonstrated that chronic treadmill exercise activates the mechanistic target of the rapamycin (mTOR) pathway in the mouse motor cortex under either naïve [15] or cocaine-exposed conditions [16]. Moreover, enhanced spinogenesis and neuronal activity were uncovered upon exercise training [5, 15, 16], depicting the neuronal modulation by aerobic exercise. It is further noticed that chronic stress also affects the mTOR pathway in the brain. For example, mTOR protein expression level was correlated with the susceptibility of mice during chronic mild stress (CMS) [17]. In another CMS mouse model, the Akt–mTOR pathway was down-regulated [18]. These results highlight the potential involvement of mTOR in exercise-mediated anxiolysis. As one of the modulatory centers for mental functions, the medial prefrontal cortex (mPFC) profoundly affects anxiety behaviors [19, 20], whilst few in vivo studies have examined the effect of exercise on mPFC activity. Moreover, axonal myelination has been correlated with cortical neuronal activity [21, 22]. Taken together, we hypothesized that exercise training might mediate the mTOR pathway in mPFC to affect neural activity for the relief of anxiety disorders.

To test this hypothesis, we generated a chronic restrain stress (CRS) model on adolescent mice, which presented anxiety-like behaviors in association with depressed mPFC neuronal activity. Fourteen-day treadmill exercise effectively prevented the anxiety phenotypes via potentiating mPFC neurons, which were dependent on cell-autonomous activation of the mTOR pathway. Moreover, axonal myelination was protected by mTOR pathway-mediated neuronal activation under exercise training. Further molecular dissections identified the role of Fragile X mental retardation protein (FMRP), which negatively affected the mTOR pathway for controlling cortical activity. Lastly, FMRP expression was found to be regulated by exercise-potentiated mRNA methylation in mPFC as we previously reported [23]. In sum, this FMRP-mTOR pathway shed new insights for illustrating the anxiolytic effects of exercise.

## Materials and methods

### Animals

All mice (4~6 weeks old, male C57BL/6J) were purchased at the Guangdong Provincial Experimental Animal Center. Animals were group-housed with food and water ad libitum. A normal light–dark phase was adopted. All mice were grouped housed (4–6 mice per cage). All animal experiments were approved by the Animal Care and Use Committee of Jinan University, in accordance with animal research guidelines stipulated by the university.

To generate a chronic restraint stress (CRS) model, mice were subjected to a restraint tube for 3 h (from 7 p.m. to 10 p.m.) every day for 14 consecutive days. The treadmill exercise was executed from 9 a.m. to 10 a.m. for 14 days. The velocity was set at 10 m per minute.

For intraperitoneal injection, 5-Bromo-2-deoxyuridine (BrdU) was administrated at 50 mg/kg daily during the last 5 days. Rapamycin was administrated every 3 days (150 mg/kg) during 2-week exercise training. K255a (10 μg/kg) was injected daily.

### Behavioral assays

To minimize the carryover effects from the previous behavioral assay, the sequence of behavioral assays were arranged from the least stressful assay (open field test), followed by the marble burying, and concluded with the elevated plus-maze with stress stimuli by the elevated platform. The 4-h interval was set between each assay and all experiments were conducted during the light phase.

#### Open field test

The test was carried out using a rectangular plastic chamber (40 × 40 × 30 cm) lighted with 25 W halogen bulbs (180 cm above). Before the experiment, the mouse was placed in the test room for 30 min habituation. The assay started with the mouse being gently placed in the central region of the field. During 5 min free movement, the trajectory of the mouse was captured by the camera and analyzed with EthoVision v7.0 package for data analysis.

#### Elevated plus maze test

Prior to the test, the mouse was placed in the test room for 30 min habituation. The assay was initiated with the gentle placement of the mouse into the central area (5 × 5 cm) of the maze. The animal was allowed to freely explore freely both open-arm (30 × 5 cm) and closed-arm regions (30 × 5 cm, with 15 cm height walls). The movement path was tracked and analyzed by EthoVision v7.0 package.

#### Marble burying test

The test was carried out in a standard cage (17.5 × 10 × 5.5 inches) which was filled with soft beddings (3 cm depth). A total of 15 glass marbles (1.4 cm diameter, dark glass) were evenly distributed in a 3 × 5 grid on the bedding. The mouse was placed into the corner of the cage and the number of marbles buried (which was defined as 2/3 or more surface covered with bedding) was recorded during 10 min trial. Video records were also kept to help with data analysis. Three trials were conducted for one animal and the results were averaged.

### RNA extraction and qPCR

Total RNA was extracted from mouse mPFC tissues using Triton Reagent (BBI) following the manual instruction. Extracted RNA was quantified on the NanoDrop (Thermo Fisher). Using mRNA as the template, cDNA was synthesized using the High-Capacity cDNA Reverse Transcription kit (TaKaRa). For qPCR, cDNA samples were mixed with TB Green Premix Ex Taq (TaKaRa) and specific primers (*Fmr1*: Forward, CAATG GCGCT TTCTA CAAGG C; Reverse, TCTGG TTGCC AGTTG TTTTC A; *Tsc2*: Forward, TGCCG CAGCA TCAGT GTATC; Reverse, TGCCA GGAGG AACTC TCCC; *Raptor*: Forward, TTTGT CTACG ACTGT TCCAA TGC; Reverse, GCTAC CTCTA GTTCC TGCTC C; *Gapdh*: Forward, AGGTC GGTGT GAACG GATTT G; Reverse, TGTAG ACCAT GTAGT TGAGG TCA). *Gapdh* gene was used as the internal reference. Quantitative RT-PCR was carried out in a CFX384 Real-Time System (Bio-Rad). Data analysis followed the 2^-△△^ approach to calculate the CT value.

### Protein extraction and Western blotting

mPFC tissues were kept in a buffer solution containing phosphatase inhibitors and protease inhibitors (Beyotime, Shanghai, China) to prepare a cell suspension. The protein solution was collected by centrifuge (1200×*g*, 10 min). The protein concentration was quantified by the BCA kit (Beyotime, Shanghai, China) following the manual instruction. Before blotting assays, the protein solution was diluted and boiled at 100 °C for 10 min. Protein samples were separated by SDS–PAGE under 80 V and 120 V electric fields. After transferring the PVDF membrane, 5% fetal bovine serum (BSA) was applied for 2 h blocking, followed by primary antibody incubation at 4 °C overnight. The membrane was subsequently washed with 0.01% TBST and incubated with a secondary antibody for 2 h. Protein bands were visualized using an imaging system (Bio-Rad, Hercules, USA), Integrated gray values of each band were measured using ImageJ (National Institutes of Health, Bethesda, USA).

### Immunofluorescent imaging

Mice were deeply anesthetized and were perfused with saline and PFA. The whole brain was quickly removed and was fixed in PFA overnight. Dehydration was then performed using 30% sucrose. The brain was prepared into 40 μm coronal slices using a sliding microtome (Leica, Germany). The brain slices were washed with 0.01% PBS, blocked with BSA for 2 h, and incubated with primary antibody (see Table S1 for details) at 4 °C for 48 h. After incubation with a secondary antibody conjugated with fluorophores, images were captured with a laser confocal microscope (Zeiss, Germany). The fluorescence intensity was analyzed using ImageJ. For morphometric study, 3 consecutive slices were analyzed and results were averaged.

### Stereotaxic injection

Mice were anesthetized with 1.25% Avertin. An incision was made on the scalp and local sterilization was performed and the periosteum tissue was gently removed. The prelimbic region of mPFC (AP: 2.33 mm; ML: ± 0.1 mm; DV: −1.2 mm) was located using a stereotaxic instrument (RWD, China). An injection hole was prepared on the skull by a high-speed micro-drill (OmniDrill35, WPI, USA). A total of 100 nl viral vectors (see Table S1 for details, using a viral titer at 10^12^ GC/ml) were slowly injected into the mPFC via a glass micropipette connected to an ultra-micro injection pump (Nanoliter 2010, World Precision Instruments, USA). The pipette was retained in the brain tissue for 8 min after the injection and was then slowly retracted. The brain skin was sutured and the mouse was returned to the home cage for recovery.

### In vivo calcium imaging

At 24 h before the imaging session, the mouse was anaesthetized to open the scalp. The skull was exposed to remove connective tissues. Then a customized metal scaffold was fixed on the skull using dental hydrogel for subsequent fixation under the microscope. On an experimental day, a high-speed drill was used to make an imaging window (2 mm length) across the skull. The skull was carefully detached with the epidural retained. A small glass slide was glued onto the skull to prepare an imaging window

Image acquisition in time-series mode was performed using with a speed of 2 Hz using an LSM780 two-photon microscope (Zeiss, Germany), with the laser power tuned below 25 mW at 920 nm excitation wavelength. The captured images were compensated using the Turbo Reg tool in the ImageJ software. Designated regions of interest (ROI) covering a somatic region of pyramidal neurons were selected to collect fluorescent (*F*) values. The Δ*F*/*F*_0_ was calculated as (*F*−*F*_0_)/*F*_0_, where the *F*_0_ was averaged *F* values during the first 10% recording period as the basal level. A calcium transient was defined when the Δ*F*/*F*_0_ was higher than threefold the standard deviation of the sample data.

### Transmission electron microscopy

To prepare the ultra-thin brain sections, the whole brain tissue of the mouse was removed and immersed in solutions (G1102, Servicebio, USA) at 4 °C for 2 h, followed by fixation with 1% osmium tetroxide for 2 h. The brain tissue was then dehydrated by gradient concentration of alcohol (50–100%, 15 min each time) and propanol (15 min twice). The coating was performed in an oven at 60 °C for 48 h using the agent (EMbed 812, SPI, USA). The ultrathin slice (80 nm) was prepared by a slicer (Leica, Germany). The prepared slices were stained with uranyl acetate in 100% alcohol for 15 min and in lead citrate for 15 min. After airdry, images were captured by transmission electron microscopy (Hitachi, Japan). Using ×13,500 magnification, axons were imaged and further measurements were performed by Image J software.

### Statistical analysis

All data were first tested for normality. For those that fitted the normal distribution, the Student’s *t*-test and one-way analysis of variance (ANOVA) were used for the comparison between two groups or multiple datasets, respectively. Tukey post hoc test was employed to compare means between two specific groups after ANOVA. For those data that did not fit the normal distribution such as those in calcium imaging, the nonparametric Kruskal–Wallis test was adopted, followed by Dunn’s post hoc comparison. The variation within each sample was estimated to be at similar levels. All data plots represent independent samples from different animals, without technical replicates. All experiments were duplicated or triplicated for the consistency of results. The sample size was estimated based on recent literature in the same field. Animals were evenly allocated into experimental groups, even though no systematic randomization approach was used. Experimenters were blinded to the grouping when analyzing behavioral and calcium recording data. The exact *N* number was identified in the figure legends. A significant level was defined when *P* < 0.05, All statistical analysis was performed by GraphPad Prism 8.0 (USA).

## Results

### Exercise prevented anxiety-like behaviors and axonal demyelination under stress

**Fig. 1:**
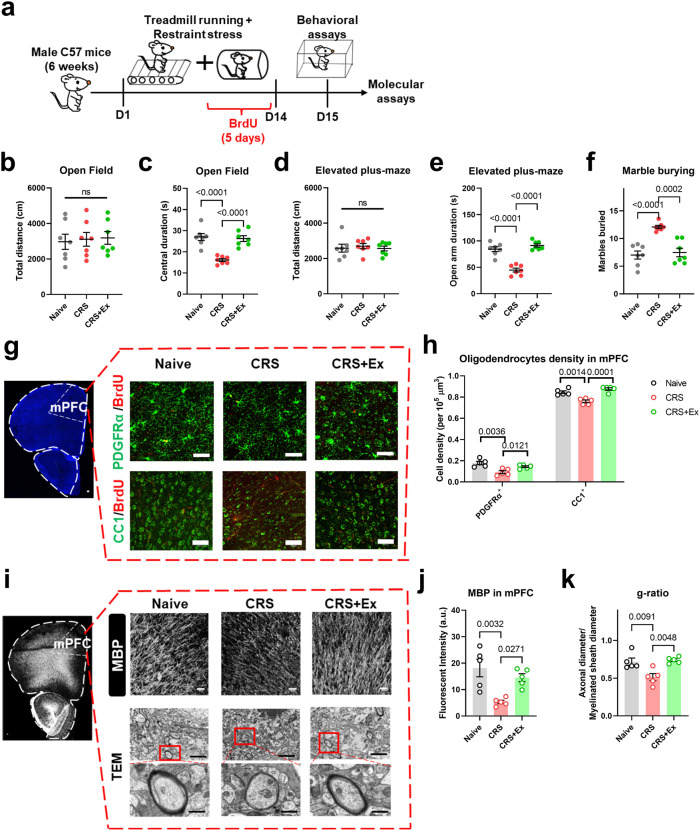
Physical exercise prevents chronic stress-induced anxiety-like behaviors and axonal demyelination. **a** Schematic illustration of experimental designs. **b** Total distance traveled in the open field was unaffected by chronic restrain stress (CRS) or exercised (Ex). One-way ANOVA, *F*(2,18) = 0.07908, *P* = 0.9243. **c** Time spent in the central region in the open field was decreased by CRS and was elevated in the Ex group. One-way ANOVA, *F*(2,18) = 21.95, *P* < 0.0001. **d** Total movement distance on the elevated plus maze was unchanged across groups. One-way ANOVA, *F*(2,18) = 0.1719, *P* = 0.8434. **e** Time duration in the open arm on the elevated plus maze was decreased by CRS and was maintained in CRS + Ex group. One-way ANOVA, *F*(2,18) = 44.95, *P* < 0.0001. **f** The number of marbles buried was increased by CRS and was suppressed in CRS + Ex group. One-way ANOVA, *F*(2,18) = 19.63, *P* < 0.0001. *N* = 7 mice in each group in (**b–f**). Tukey’s post-hoc test was used for comparisons between the two groups. **g** Fluorescent images showing the co-labeling of oligodendrocyte marker (PDGFRα or CC1) with BrdU. Scale bar, 100 μm. **h** The density of PDGFRα or CC1 cells was decreased by CRS and was maintained in CRS + Ex group. Multiple *t*-test was used for comparisons between the two groups. **i** Upper panels, fluorescent images of MBP in mPFC region. Middle and lower panels, transmission electron microscopy (TEM) images of axonal fibers, with high-magnification images showing the morphology of individual myelin sheath (dark shade). Scale bar, 100 μm in upper panels, 2 μm in middle panels, and 500 nm in lower panels. **j** Fluorescent intensity (in arbitrary unit, or a.u.) of MBP was decreased by CRS and was maintained in CRS + Ex group. One-way ANOVA, *F*(2,12) = 9.436, *P* = 0.0034. **k** The g-ratio (defined as the diameter of unmyelinated axonal fibers divided by the diameter of the myelinated sheath) was decreased by CRS and was maintained in CRS + Ex group. One-way ANOVA, *F*(2,12) = 9.691, *P* = 0.0031. *N* = 5 mice in each group in (**g–k**). Tukey’s post-hoc test was used for comparisons between the two groups. All data were presented as mean ± sem.

We at first generated a CRS model on male adolescent mice (5–6 weeks) to replicate anxiety-like behaviors, whilst the general locomotor behaviors remained unaffected (Fig. 1a–f), in addition to unchanged depressive behaviors (Fig. S1). Two-week treadmill exercise (1 h daily) effectively prevented the occurrence of these anxiety phenotypes (Fig. 1c, e, and f). In searching for the neural substrate underlying those behavioral changes, BrdU was injected for tracking the *de novo* cell proliferation inside mPFC. Histological quantification did not reveal significant changes in newly formed astrocytes (GFAP+) or microglial cells (Iba1+; Fig. S2). The magnitude of oligodendrogenesis, however, was remarkably suppressed by CRS and was maintained under exercise (Fig. S2). In specific, both precursors (PDGFRα+) and mature oligodendrocytes (CC1+) showed elevated densities in the exercise group (Fig. 1g, h), supporting the protection of normal oligodendrogenesis. Consequently, the structural integrity of axonal myelin sheath was maintained under the treadmill paradigm, as suggested by higher expression of myelin basic protein (MBP; Fig. 1i, j) and increased g-ratio using transmission electron microscopy (TEM; Fig. 1i, k). These data collectively suggested the prevention of anxiety disorders and axonal demyelination by exercise training.

### Exercise activates brain mTOR pathway to maintain axonal myelination for conferring stress resilience in adolescent mice

**Fig. 2:**
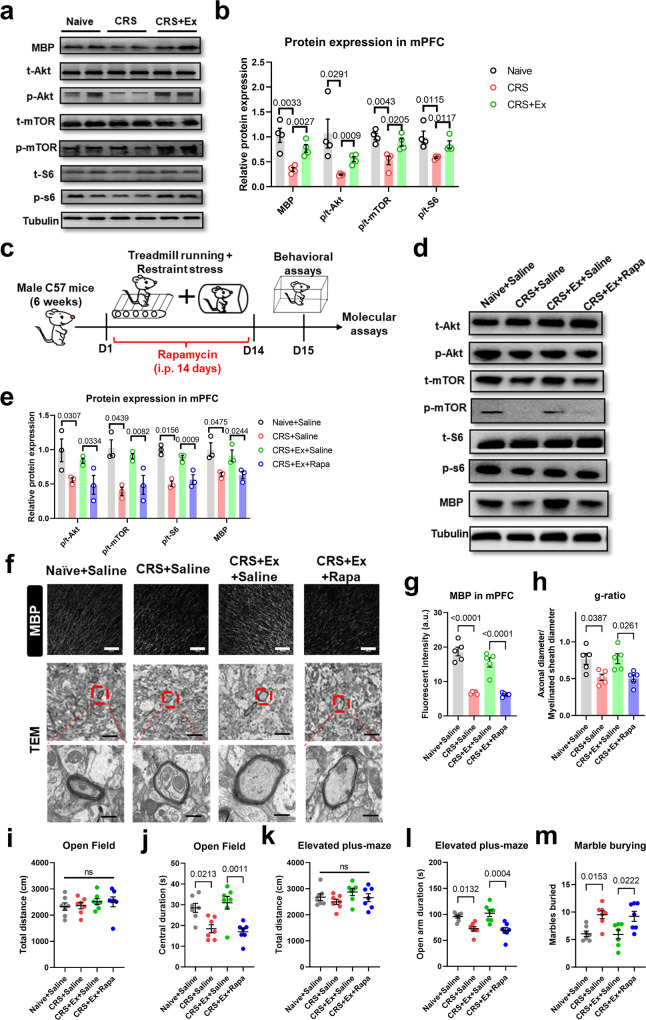
Exercise potentiates mTOR pathway for anxiolysis and myelination. **a** Representative blotting images for MBP and proteins in the mTOR pathway. **b** Quantification of relative protein expression levels. MBP, p-Akt, p-mTOR and p-S6 were all downregulated by CRS and were upregulated in CRS + Ex group. Multiple *t*-test was used for comparisons between the two groups. *N* = 4 mice per group. **c** Experimental design for the mTOR-inhibition assay, during which rapamycin (Rapa, 150 mg/kg daily) was injected into exercised mice. **d** Western blotting images of Akt-mTOR pathway and MBP after rapamycin infusion. **e** Quantification of proteins showed the inhibition of the mTOR pathway and downregulation of MBP by rapamycin injection. *N* = 3 mice per group. Multiple *t*-test was used for comparisons between the two groups. **f** Upper panels, fluorescent images of MBP in mPFC region. Middle and lower panels, TEM images of axonal fibers, with high-magnification images showing the morphology of individual myelin sheath (dark shade). Scale bar, 100 μm in upper panels, 2 μm in middle panels, and 500 nm in lower panels. **g** Fluorescent intensity (in a.u.) of MBP was decreased by rapamycin injection. One-way ANOVA, *F*(3,16) = 44.80, *P* < 0.0001. **(h)** The g-ratio was decreased in CRS + Ex+Rapa group. One-way ANOVA, *F*(3,16) = 6.392, *P* = 0.0047. *N* = 5 mice in each group in (**g** and **h**). Tukey’s post-hoc test was used for comparisons between the two groups. **i** Total distance traveled in the open field was unaffected by rapamycin injection. One-way ANOVA, *F*(3,24) = 0.4764, *P* = 0.7016. **j** Time spent in the central region in the open field was decreased by rapamycin injection. One-way ANOVA, *F*(3,24) = 9.695, *P* = 0.0002. **k** Total distance on the elevated plus maze remained unchanged in CRS + Ex+Rapa group. One-way ANOVA, *F*(3,24) = 1.389, *P* = 0.2701. **l** Time duration in the open arm on the elevated plus maze was decreased in CRS + Ex+Rapa group. One-way ANOVA, *F*(3,24) = 11.34, *P* < 0.0001. **m** The number of marbles buried was increased by rapamycin. One-way ANOVA, *F*(3,24) = 5.181, *P* = 0.0067. *N* = 7 mice in each group in (**i–m**). Tukey’s post-hoc test was used for comparisons between the two groups. All data were presented as mean ± sem.

Next, we investigated the molecular mechanism governing anxiolysis by exercise training. Our previous findings presented the activation of mTOR upon endurance training [15, 16], and the pivotal role of mTOR in oligodendrogenesis and myelination has been reported [24, 25]. Western blotting assays found that, in association with elevated MBP, the phosphorylation level of mTOR core protein was decreased under CRS and was potentiated by exercise (Fig. 2a, b). At the upstream and downstream of mTOR, the level of phosphorylated Akt and ribosomal protein S6 were all increased after exercise training (Fig. 2a, b). To further demonstrate the causal relationship between the mTOR pathway and exercise-mediated myelination, we infused those mice with rapamycin during the treadmill training period (Fig. 2c). The inhibition of the mTOR pathway in exercised mice remarkably decreased MBP expression in mPFC (Fig. 2d, e). Such demyelination status can be attributed to a decreased level of oligodendrogenesis upon mTOR inactivation (Fig. S3). As the consequence, the density of myelinated axons as well as the relative thickness of myelin sheath (g-ratio) were all decreased after rapamycin injection, largely antagonizing the recovery effect of exercise training (Fig. 2f–h). Lastly, the anxiolytic effect of treadmill exercise was also eliminated by mTOR inhibition, although the overall locomotor was unchanged (Fig. 2i–m). These results converged to imply the contribution of the mTOR pathway to exercise-mediated stress resilience and axonal myelination.

**Fig. 3:**
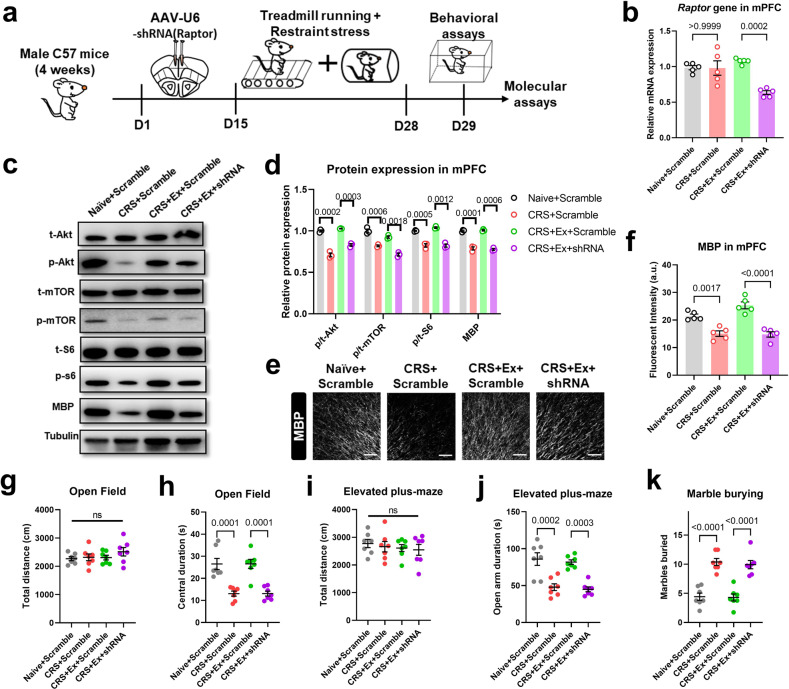
Brain mTOR pathway mediates exercise effect on anxiety and myelination. **a** Schematic illustration for experimental designs of mPFC-specific mTOR inhibition. **b** Relative mRNA expression of the *Raptor* gene showed the effective knockdown by shRNA. One-way ANOVA, *F*(3,16) = 12.48, *P* = 0.0002. *N* = 5 mice per group. Tukey’s post-hoc test was used for comparisons between the two groups. **c** Representative Western blotting bands for mTOR proteins and MBP expression in PFC extracts. **d** Quantification of relative protein expression showed the suppression of p-Akt, p-mTOR, p-S6, and MBP proteins when the *Raptor* gene was knocked down in mPFC. Multiple *t*-test was used for comparisons between the two groups. *N* = 3 mice per group. **e** Fluorescent images of MBP in the mPFC region showed the demyelination under *Raptor* gene knockdown. Scale bar, 100 μm. **f** Fluorescent intensity (in a.u.) of MBP was decreased after *Raptor* gene knockdown. One-way ANOVA, *F*(3,16) = 26.84, *P* < 0.0001. *N* = 5 mice per group. Tukey’s post-hoc test was used for comparisons between the two groups. **g** Total distance traveled in the open field was unaffected under *Raptor* gene knockdown. One-way ANOVA, *F*(3,24) = 1.002, *P* = 0.4090. **h** Time spent in the central region in the open field was decreased under *Raptor* gene knockdown. One-way ANOVA, *F*(3,24) = 18.47, *P* < 0.0001. **i** The locomotor activity on the elevated plus maze was unaffected in CRS + Ex+shRNA group. One-way ANOVA, *F*(3,24) = 0.3355, *P* = 0.7998. **j** Time duration in the open arm on the elevated plus maze was decreased in CRS + Ex+shRNA group. One-way ANOVA, *F*(3,24) = 16.48, *P* < 0.0001. **k** The number of marbles buried was increased by suppressing *Raptor* gene expression. One-way ANOVA, *F*(3,24) = 27.90, *P* < 0.0001. *N* = 7 mice in each group in (**g–k**). Tukey’s post-hoc test was used for comparisons between the two groups. All data were presented as mean ± sem.

The administration of rapamycin potentially inhibited the mTOR pathway in both central and peripheral tissues. To further identify the brain specificity, we adopted an adeno-associated virus (AAV)-mediated vector expression of the short hairpin RNA (shRNA) to knockdown the expression of one of the core mTOR proteins, Raptor (Fig. 3a, b). Such mPFC-specific genetic manipulation effectively suppressed the local mTOR pathways (Fig. 3c, d) and decreased MBP levels (Fig. 3e, f). In a similar manner as those of rapamycin infusion, genetic knockdown of *Raptor* in mPFC abolished the exercise-mediated anxiolytic effects (Fig. 3g–k), illustrating the necessary role of the brain mTOR pathway. On the other hand, we further demonstrated the sufficiency of activating the local mTOR pathway in conferring stress resilience by suppressing the expression of upstream inhibitor Tsc2 (Fig. S4a, b), leading to potentiated mTOR phosphorylation and MBP expression in CRS mice (Fig. S4c–f). It is worth noticing that no significant effect on mTOR activity or MBP occurred in naïve mice with *Tsc2* gene interference (Fig. S4c–f). These results implied that the homeostasis of mTOR was only disrupted upon CRS and can be recovered by exercise training. Finally, mTOR activation mimicked the anxiolytic effects of treadmill training on CRS mice (Fig. S4g–k). In one word, endurance training activates the brain mTOR pathway, which prevents demyelination and anxiety-like behaviors in CRS-treated mice.

### Neuronal activity was mediated by mTOR to affect axonal myelination

**Fig. 4:**
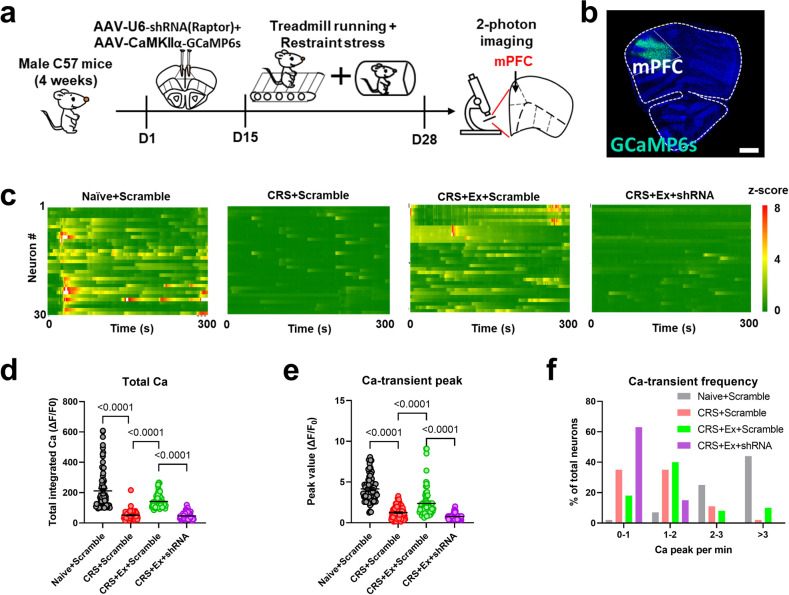
Brain mTOR pathway regulates cortical neuronal activity. **a** Experimental flowchart of in vivo 2-photon imaging assay. **b** Expression of GCaMP6s in mPFC region. Scale bar, 500 μm. **c** Heatmaps showing the in vivo calcium activities of mPFC neurons. A total of 30 neurons were displayed, with the calcium transients transformed as *z*-scores. **d** Total integrated calcium levels of mPFC were decreased by CRS and re-elevated by exercise training. The inhibition of the mTOR pathway repressed calcium activities in exercised mice. Nonparametric Kruskal–Wallis test statistic = 234.4, *P* < 0.0001. **e** Similar trends were observed for the peak values of calcium transients. Nonparametric Kruskal–Wallis test statistic = 198.1, *P* < 0.0001. **f** Frequency distribution of calcium transient frequency (spike per min) also showed lower frequency by *Raptor* gene knockdown. *n* = 80 neurons from 4 mice in each group in (**d–f**). Dunn’s test was used for the comparison between the two groups. All data were presented as mean ± sem.

Previous knowledge has established the relationship between mTOR and neuronal activity in the mouse motor cortex [15, 16]. We thus tested if similar mechanisms exist in mPFC for regulating mental functions. By co-transfecting shRNA(Raptor) along with genetically coded fluorescent calcium indicator GCaMP6s into mPFC, we utilized 2-photon in vivo calcium imaging to capture the activity of mPFC neurons (Fig. 4a, b). The recording at single-cell resolution showed that exercise effectively maintained normal calcium activity in CRS mice, whilst genetically knocking down the *Raptor* gene abolished such effects (Fig. 4c, d). Quantitative analysis revealed that exercise potentiated both peak values and frequency of calcium transients of cortical neurons (Fig. 4e, f). On the other hand, the role of mTOR activation in neural activity was also tested via repressing *Tsc2* gene expression (Fig. S5a, b). The disinhibition of the mTOR pathway did not affect neuronal activity in naïve mice but augmented total calcium transients in CRS-treated ones (Fig. S5c–f). These results suggest the bidirectional modulation of neural activity by the mTOR pathway in a cell-autonomous manner.

**Fig. 5:**
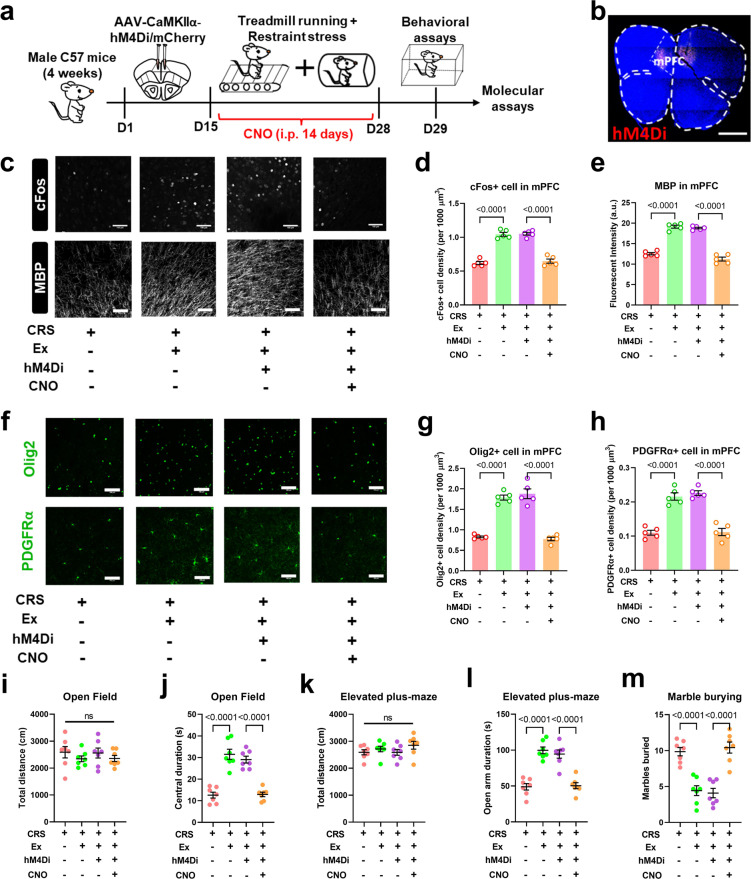
mPFC neuronal activity mediates axonal myelination under the exercise paradigm. **a** Experimental designs of chemogenetic inhibition assay. **b** Infection sites of AAV-hM4Di into mPFC. Scale bar, 500 μm. **c** Fluorescent images showing cFos (upper panels) and MBP (lower panels). Scale bar, 100 μm. **d** Quantification of cFos suggested the potentiation of mPFC neurons under exercise training and neuronal inhibition by CNO infusion. One-way ANOVA, *F*(3,16) = 87.31, *P* < 0.0001. **e** MBP fluorescent intensity (in a.u.) was depressed under neuronal inhibition. One-way ANOVA, *F*(3,16) = 143.3, *P* < 0.0001. **f** Fluorescent intensity of oligodendrocytes (Olig2 and PDGFRα) in mPFC. Scale bar, 100 μm. **g** Quantification showed the decreased density of Olig2+ cells after chronic inhibition of mPFC neurons. One-way ANOVA, *F*(3,16) = 71.57, *P* < 0.0001. **h** The density of PDGFRα cells was decreased in CNO-treated animals. One-way ANOVA, *F*(3,16) = 49.70, *P* < 0.0001. *N* = 5 mice in each group in (**c–h**). Tukey’s post-hoc test was used for comparisons between the two groups. **i** Total distance in the open field was unaffected under mPFC neuron inhibition. One-way ANOVA, *F*(3,24) = 0.6556, *P* = 0.5873. **j** Time spent in the central region in the open field was decreased under mPFC neuron inhibition. One-way ANOVA, *F*(3,24) = 37.99, *P* < 0.0001. **k** Total locomotor activity on the elevated plus maze was unchanged in CRS+Ex+hM4D+CNO group. One-way ANOVA, *F*(3,24) = 1.188, *P* = 0.3351. **l** Time duration in the open arm on the elevated plus maze was decreased in CRS+Ex+hM4D+CNO group. One-way ANOVA, *F*(3,24) = 32.61, *P* < 0.0001. **m** The number of marbles buried was increased by CNO treatment. One-way ANOVA, *F*(3,24) = 25.50, *P* < 0.0001. *N* = 7 mice in each group in (**i–m**). Tukey’s post-hoc test was used for comparisons between the two groups. All data were presented as mean ± sem.

Due to the established linkage between neuronal activity and axonal myelination in the motor cortex [21], it is interesting to test if neuronal activity in mPFC also affects the structural plasticity of the local myelin sheath. We applied the chemogenetics approach via transfecting the designer receptors exclusively activated by designer drugs (DREADD) receptor hM4Di into bilateral mPFC of exercised mice. The application of specific ligand clozapine-N-oxide (CNO) largely repressed the activity of mPFC neurons (Fig. 5a–d). Of note, the expression level of MBP was also decreased (Fig. 5c, e). The axonal demyelination can be attributed to the lower number of precursor cells or differentiated oligodendrocytes (Fig. 5f–h). Consequently, the anxiolytic effect of exercise on CRS mice was also abolished under neuronal inhibition (Fig. 5i–m). These results suggested the necessity of mPFC activation in exercise-mediated axonal myelination. Moreover, the mPFC neuronal population was activated using excitatory chemogenetic receptor hM3Dq, which largely replicated exercise effects including elevated MBP expression, higher density of oligodendrocytes and precursor cells, as well as decreased anxiety-like behaviors (Fig. S6). Our data thus collectively demonstrated the activity-dependent axonal myelination and anxiolysis by exercise training via activating the brain mTOR pathway.

### RNA methylation affects FMRP to modulate mTOR activity in the exercised brain

**Fig. 6:**
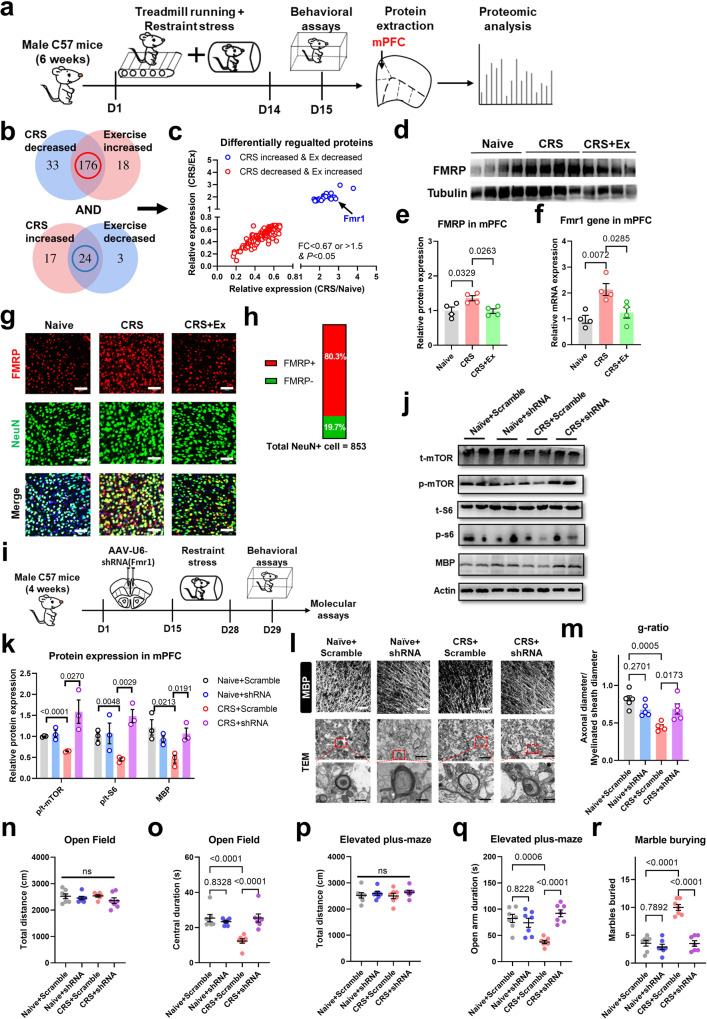
FMRP underlies exercise-mediated stress resilience and myelination. **a** Experimental design for the proteomic study. **b** Venn diagrams showing the number of differentially regulated genes (DEGs) by both CRS and exercise intervention. **c** Plotting for those 200 DEGs in **b** with their fold changes (FCs) of expression. **d** Representative blotting bands for FMRP in mPFC extracts. **e** Quantification analysis showed elevated FMRP expression under CRS and depression by exercise. One-way ANOVA, *F*(2,9) = 5.337, *P* = 0.0271. **f** The mRNA level of the *Fmr1* gene was also upregulated by CRS and downregulated under exercise. One-way ANOVA, *F*(2,9) = 9.904, *P* = 0.0069. *N* = 4 mice in each group in (**d–f**). Tukey’s post-hoc test was used for comparisons between the two groups. **g** Immunofluorescent labeling for FMRP in neurons (NeuN + ). Scale bar, 100 μm. **h** Percentage of NeuN+ cells expressing FMRP. **i** Schematic illustrations for experiments of *Fmr1* gene knockdown. **j** Representative blotting bands for mTOR and MBP proteins after *Fmr1* knockdown. **k** Quantifications showed the reactivation of the mTOR pathway and elevated MBP expression in CRS mice under *Fmr1* gene silence. Multiple *t*-test was used for comparisons between the two groups. *N* = 3 mice per group. **l** Upper panels, fluorescent images of MBP in mPFC region. Middle and lower panels, TEM images of axonal fibers, with high-magnification images showing the morphology of myelin sheath (dark shade). Scale bar, 100 μm in upper panels, 2 μm in middle panels, and 500 nm in lower panels. **m** The g-ratio was re-elevated after the *Fmr1* gene knockdown. One-way ANOVA, *F*(3,16) = 9.323, *P* = 0.0008. *N* = 5 mice in each group. Tukey’s post-hoc test was used for comparisons between the two groups. **n** Total distance traveled in the open field remained unchanged under *Fmr1* gene knockdown. One-way ANOVA, *F*(3,24) = 1.059, *P* = 0.3850. **o** Time spent in the central region in the open field was increased under *Fmr1* gene knockdown. One-way ANOVA, *F*(3,24) = 13.44, *P* < 0.0001. **p** Locomotor activity on the elevated plus maze was unaffected in CRS + shRNA group. One-way ANOVA, *F*(3,24) = 0.5513, *P* = 0.6522. **q** Time duration in the open arm on the elevated plus maze was increased in CRS + shRNA group. One-way ANOVA, *F*(3,24) = 12.08, *P* < 0.0001. **r** The number of marbles buried was decreased by suppressing *Fmr1* gene expression. One-way ANOVA, *F*(3,24) = 43.59, *P* < 0.0001. *N* = 7 mice in each group in (**n–r**). Tukey’s post-hoc test was used for comparisons between the two groups. All data were presented as mean ± sem.

Lastly, we investigated the molecular mechanisms governing mTOR activity in exercised mice. Using a proteomic approach to analyze expressional profiles in mPFC (Fig. 6a), we identified a total of 200 proteins that were significantly regulated by both CRS and exercise intervention (Fig. 6b). Further analysis discovered that the *Fmr1* gene, which encodes Fragile X mental retardation protein (FMRP), was remarkably increased by CRS and was decreased by exercise training (Fig. 6c–f). Due to the documented role of FMRP in inhibiting the mTOR pathway [26], we tested if FMRP directs mTOR activity under CRS paradigms. As FMRP was mainly expressed in mPFC neurons (Fig. 6g, h), we introduced shRNA targeting the *Fmr1* gene under an AAV vector (Fig. 6i). Molecular assays found the re-activation of the mTOR pathway by FMRP downregulation in CRS animals, whilst no significant change was observed on naïve mice (Fig. 6j, k). These patterns largely recapitulated exercise-mediated effects. In consistent with molecular changes, we also found the potentiation of neuronal activities via in vivo calcium imaging (Fig. S7). Consequently, axonal myelination was protected in CRS mice when the *Fmr1* gene was knocked down (Fig. 6l, m). Further behavioral phenotyping showed the prevention of anxiety-like behaviors caused by CRS upon *Fmr1* gene silence (Fig. 6n–r). Such an FMRP–mTOR axis probably explained the axonal demyelination and anxiety-like behaviors under CRS, as well as the exercise-mediated anxiolytic effects.

**Fig. 7:**
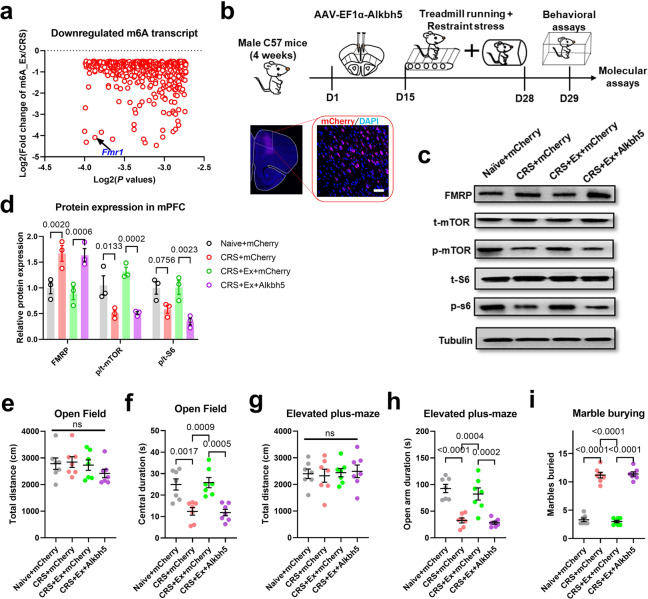
Exercise-mediated *Fmr1* transcript methylation to activate mTOR pathway. **a** The plotting of exercise-mediated m6A transcript, in contrast with the m6A level in the CRS group. **b** Experimental design for RNA methylation interference using *Alkbh5* gene overexpression. **c** Representative blotting bands for FMRP, p-mTOR and p-S6. **d** Quantification analysis found that blocking RNA methylation in exercised mice elevated FMRP expression and suppressed the mTOR pathway. Multiple *t*-test was used for comparisons between the two groups. *N* = 3 mice per group. **e** Total distance traveled in the open field was unaffected. One-way ANOVA, *F*(3,24) = 0.9731, *P* = 0.4217. **f** Time spent in the central region in the open field was decreased with *Alkbh5* gene overexpression. One-way ANOVA, *F*(3,24) = 13.09, *P* < 0.0001. **g** The general locomotor activity on the elevated plus maze was unchanged. One-way ANOVA, *F*(3,24) = 0.1128, *P* = 0.9518. **h** Time duration in the open arm on the elevated plus maze was decreased in CRS+Ex+Alkbh5 group. One-way ANOVA, *F*(3,24) = 20.14, *P* < 0.0001. **i** The number of marbles buried was increased by interfering RNA methylation. One-way ANOVA, *F*(3,24) = 160.7, *P* < 0.0001. *N* = 7 mice in each group in (**e–i**). Tukey’s post-hoc test was used for comparisons between the two groups. All data were presented as mean ± sem.

The last question is how exercise training modulated the FMRP–mTOR pathway. As one lifestyle intervention, aerobic exercise primarily affects body metabolism, which may regulate brain structure and function via specific metabolites. Our recent work, for example, has revealed the role of hepatic biosynthesis of S-adenosyl methionine (SAM) in mediating brain RNA methylation for preventing CRS-induced anxiety [23]. From previous N6-methyladenosine (m6A) immunoprecipitation-based RNA sequencing data [23], we found that the m6A status of *Fmr1* was largely affected in exercised mice (Fig. 7a), implying the possible epigenetic mechanism at the upstream of FMRP–mTOR axis. We, therefore, utilized a genetic approach to interfere with the exercise-mediated brain RNA methylation by locally overexpressing RNA demethylase gene *Alkbh5* (Fig. 7b). Molecular assays found that the disruption of RNA methylation process abolished exercise effects, including the elevation of FMRP and consequently inhibition of mTOR pathway (Fig. 7c, d). Behavioral phenotyping also revealed the blockade of the anxiolytic effect after interfering with the RNA methylation process (Fig. 7e–i). In line with these results, we also used an shRNA to target the *Alkbh5* gene in CRS mice (Fig. S8a), which showed the suppression of FMRP and activation of the mTOR pathway (Fig. S8b, c), in association with anxiolysis after demethylase gene knockdown (Fig. S8d–h). These data collectively suggest the epigenetic modulation of the FMRP–mTOR pathway.

**Fig. 8:**
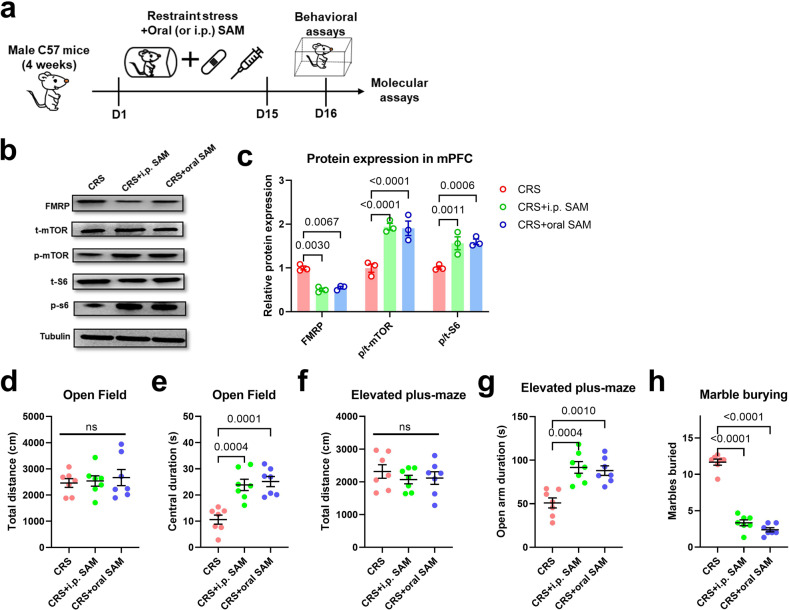
Enhancement of brain RNA methylation activates mTOR pathway to improve stress resilience. **a** Experimental design for methyl donor supplement assay. **b** Representative blotting bands for FMRP, p-mTOR and p-S6. **c** Quantification analysis found that supplements of methyl donor molecules in CRS mice suppressed FMRP expression and activated the mTOR pathway. Multiple *t-tests* were used for comparisons between the two groups. *N* = 3 mice per group. **d** Total distance traveled in the open field was unaffected. One-way ANOVA, *F*(2,18) = 0.2076, *P* = 0.8144. **e** Time spent in the central region in the open field was increased under oral or i.p. SAM. One-way ANOVA, *F*(2,18) = 17.07, *P* < 0.0001. **f** The general locomotor activity on the elevated plus maze was unchanged. One-way ANOVA, *F*(2,18) = 0.5301, *P* = 0.5975. **g** Time duration in the open arm on the elevated plus maze was increased in CRS+i.p. or oral SAM group. One-way ANOVA, *F*(2,18) = 14.14, *P* = 0.0002. **h** The number of marbles buried was decreased by enhancing RNA methylation. One-way ANOVA, *F*(2,18) = 186.3, *P* < 0.0001. *N* = 7 mice in each group in (**d–h**). Tukey’s post-hoc test was used for comparisons between the two groups. All data were presented as mean ± sem.

In previous work, we have demonstrated that oral or intraperitoneal supplements of methyl donor SAM effectively maintained normal brain RNA methylation network [23], we thus explored if FMRP–mTOR pathway was also involved. After the replenishment of SAM into CRS model mice (Fig. 8a), we found the suppression of FMRP and activation of the mTOR pathway (Fig. 8b, c), largely replicating exercise effects. These molecular substrates paralleled with the prevention of anxiety-like behaviors (Fig. 8d–h). In sum, the exercise-driven brain RNA methylation network suppresses the expression of FMRP, which activates the cortical mTOR pathway and contributes to neuronal activity and axonal myelination. These molecular and cellular changes ultimately direct the anxiolytic effect of exercise training in adolescent mice when facing environmental stress.

## Discussion

In the current study, we found prominent demyelination in mPFC under CRS in adolescent mice and showed the protection of normal myelin sheath by physical exercise. The under-development of axonal myelination has been reported across different stress models, especially in early-life stress (ELS) mice [27, 28]. Our work agreed with those works and discovered the prevention of stress-induced demyelination by endurance training during the adolescent stage. More importantly, an FMRP–mTOR pathway was proposed to be responsible for activating cortical neurons and maintaining normal myelination, contributing to enhanced stress resilience.

Previous works have demonstrated the relationship between axonal myelination and environmental stress. For example, the thickness of the myelin sheath in mPFC was closely associated with the resilience or sensitivity of the mouse under chronic social defeat stress [29]. The disrupted myelination in mPFC may also be related to cognitive dysfunctions in a mouse model of schizophrenia [30]. Independent lines of evidence highlighted the effect of ELS at the early postnatal stage [27, 28], during which axonal myelination is experiencing a “critical period” with high dynamics. The deformation of axonal myelination under chronic stress thus can be attributed to the impaired maturation of oligodendrocytes and their precursor cells [31]. These results converged to show that mPFC myelination is susceptible to different kinds of environmental stress and certain neurological diseases. In the current CRS model involving adolescent mice, the axonal myelin sheath retains relatively high plasticity [32], in line with our observations of the dynamic changes of myelin structures in response to various environmental stimuli.

In the recent decade, axonal myelination was found to be mediated by the activity of cortical neurons [21, 22]. Although the neuronal activity of mPFC during the early postnatal stage (P2–P14) has been shown to be critical for myelin formation and mental functions in later adulthood [28], few studies have dissected the axonal myelination pattern in the later adolescent stage in association with neural activity. Our findings provide evidence showing that neural activity can affect the plasticity of axonal myelination in mPFC during adolescence. In addition to those excitatory cells, GABAergic interneurons are critical for higher cognitive and mental functions, and their axonal myelination is related to cognitive dysfunctions [33]. GABAergic neurons such as parvalbumin (PV)-positive interneurons also presented activity-dependent myelination, thereby modulating axonal transmission [34]. Therefore, neural network activity is tightly related to axonal myelination, and the modulation of neuronal activity can reshape the plasticity of the myelin sheath. These results raise the possibility of using the neuromodulation approach to affect white matter structure for alleviating related mental disorders.

Physical exercise is one effective lifestyle intervention for neurological diseases and psychiatric disorders. Among different mechanistic models, axonal myelination is being appreciated by recent advances. In human patients, physical rehabilitation is one accepted approach for demyelination diseases such as multiple sclerosis [35, 36]. Using rodent models, exercise training can promote oligodendrocyte differentiation and myelination in hippocampal CA1 to relieve depressive behaviors [13], and help to preserve myelin density after stroke [37]. In an obesity-induced cognitive deficit mouse model, exercise helped to prevent the white matter damage, contributing to the recovery of memory deficits [38]. In this study, we identified the prevention of CRS-induced demyelination in mouse mPFC, expanding the current knowledge for exercise-related improvements of white matter structure. Moreover, the beneficiary effects of exercise training on axonal myelination have been replicated by other environmental interventions. For example, social interaction helped to improve the demyelination of an autistic mouse model [39]. We thus expect that multi-dimensional stimuli combing with sensory or social stimuli with exercise training could improve the white matter microstructure in human patients.

To provide the possible molecular mechanism of exercise-mediated myelination, different molecular pathways have been proposed including Rho kinase [40], Nogo-A [41], peroxisome proliferator-activated receptor gamma co-activator 1-alpha (PGC1α) [42] and Wnt signaling [43]. Our group previously found that the activation of the mTOR pathway probably contributed to the enhanced myelination in the mouse motor cortex under treadmill paradigms [15]. Current findings suggest the participation of the mTOR pathway across different stages of myelination including oligodendrocytes precursors proliferation [44], differentiation [45], and myelin growth [46]. Therefore, exercise-driven mTOR probably contributed to the protection against stress-induced demyelination. To support this hypothesis, we used both pharmaceutical and genetic approaches to block mTOR activity. Although rapamycin assay implied the role of mTOR activation under exercise-mediated axonal myelination, such an approach did not have tissue- or cell-specificity. We thus utilized the AAV-mediated genetic knockdown of the *Raptor* gene, which led to the abolishment of beneficiary effects of exercise training. Since the AAV vector mainly affected neurons rather than oligodendrocytes, we further proposed that the neuron–autonomous mTOR pathway may regulate axonal myelination by affecting neuronal activity.

At the upstream of mTOR, proteomic studies implied FMRP to be one of the factors responsible for exercise effects. FMRP is one widely expressed RNA binding protein that can prominently affect neuronal cytoskeleton and activity [47]. In particular, FMRP can negatively regulate mTOR activity [26], providing a possible route by which epigenetic regulation affects mTOR under exercise training. Although previous studies also suggest the necessary role of FMRP in maintaining the normal development of oligodendrocytes and myelin sheath [48, 49], the current work mainly investigated the role of FMRP in neurons. Our results agreed that the loss-of-function of the *Fmr1* gene elevated mTOR signaling in neurons [50, 51], in addition to higher activity in mPFC neurons. As an extra layer of molecular mechanism to link body endurance exercise and FMRP–mTOR pathway in cortical neurons, we re-visited the brain RNA m6A network [23] and identified the epigenetic regulation on the *Fmr1* transcript by exercise training. Current knowledge has revealed the prominent effect of *Fmr1* gene expression by DNA methylation [52]. Our data recognized FMRP regulation by RNA methylation, expanding the regulatory mechanisms of this critical protein for neural function.

Besides FMRP–mTOR pathway as demonstrated in the current work, exercise may also mediate mTOR activity via other upstream regulators. As one pivotal hub in cellular metabolism, the mTOR core is under tight regulation of multiple factors, many of which are potentially mediated by exercise training. For example, BDNF and its receptor TrkB can be activated under treadmill exercise to activate the mTOR pathway [5]. Exercise training also activates the AMP-activated protein kinase (AMPK)-mTOR signal in the brain [53]. As one potent inhibitor of mTOR, TSC2 activity was also found to be mediated by resistance exercise in human skeletal muscles [54]. In a similar manner, PTEN was also downregulated by exercise training in mouse pituitary [55]. These studies suggest that multiple targets exist under exercise schemes to target the mTOR pathway, and future works can be performed to illustrate the brain mTOR network under exercise intervention.

In summary, our results suggested an FMRP–mTOR pathway that maintained neuronal activity and axonal myelination of mPFC under exercise paradigms, providing the molecular substrate for exercise-mediated anxiolytic effects in adolescent mice. These works expand our understanding of the modulation of neural plasticity by environmental factors including stress and exercise.

## Supplementary information

Supplemental Information
