# Supplementary material for: Physical exercise mediates a cortical FMRP–mTOR pathway to improve resilience against chronic stress in adolescent mice
Source: Transl Psychiatry. 2023 Jan 19;13:16. doi: 10.1038/s41398-023-02311-x (PMC9852236; doi:10.1038/s41398-023-02311-x)
Supplement: Supplementary file 1 — Supplemental Information [file 41398_2023_2311_MOESM1_ESM.pdf]

Supplemental Information

Physical exercise mediates a cortical FMRP-mTOR pathway to improve resilience against chronic stress in adolescent mice

Fig. S1-S8

Table S1

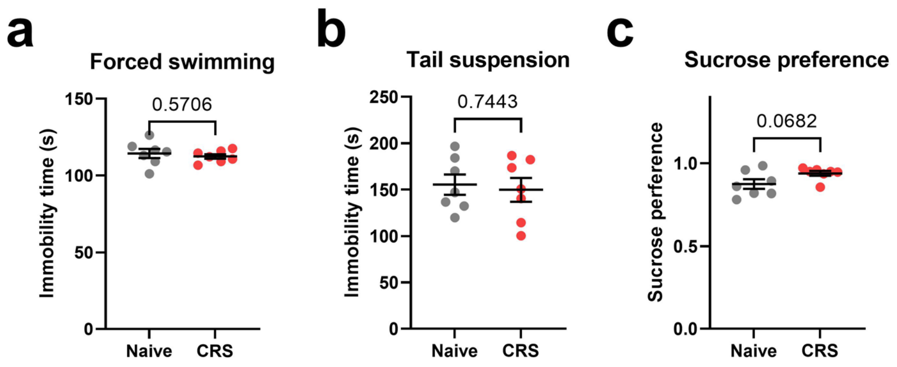

Fig. S1. No change of depressive behaviors in CRS model. Related to Fig. 1.

(a) Unchanged immobility time in the forced swimming test. Two-sample student *t*-test, *t*(12)=0.5831, *P*=0.5706.

(b) No significant difference of immobility time in the tail suspension assay. *t*(12)=0.3337, *P*=0.7443.

(c) Similar levels of sucrose preference ration between naïve and CRS mice. *t*(12)=2.003, *P*=0.0682.

N=7 mice in each group. All data were presented as mean±sem.

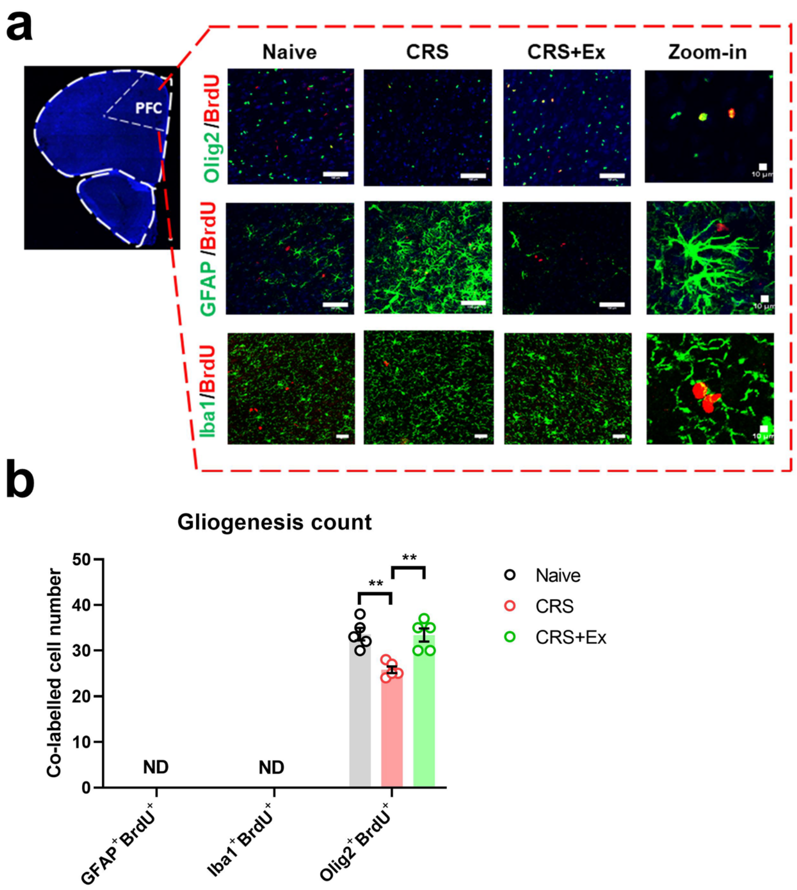

Fig. S2. Exercise potentiated oligodendrogenesis, related to Fig. 1.

(a) Fluorescent images of oligodendrocytes (Olig2+), astrocytes (GFAP+) and microglia (Iba1+) with BrdU in mOPFC. Scale bar, 100 μm or 10 μm (for zoom-in images).

(b) CRS decreased the number of newly formed Olig2+ cells and exercise training recovered oligodendrogenesis. Multiple *t*-test was used for between-group-comparison. ND, not detected; $^{**}P<0.01$. $N=5$ mice per group. All data were presented as mean±sem.

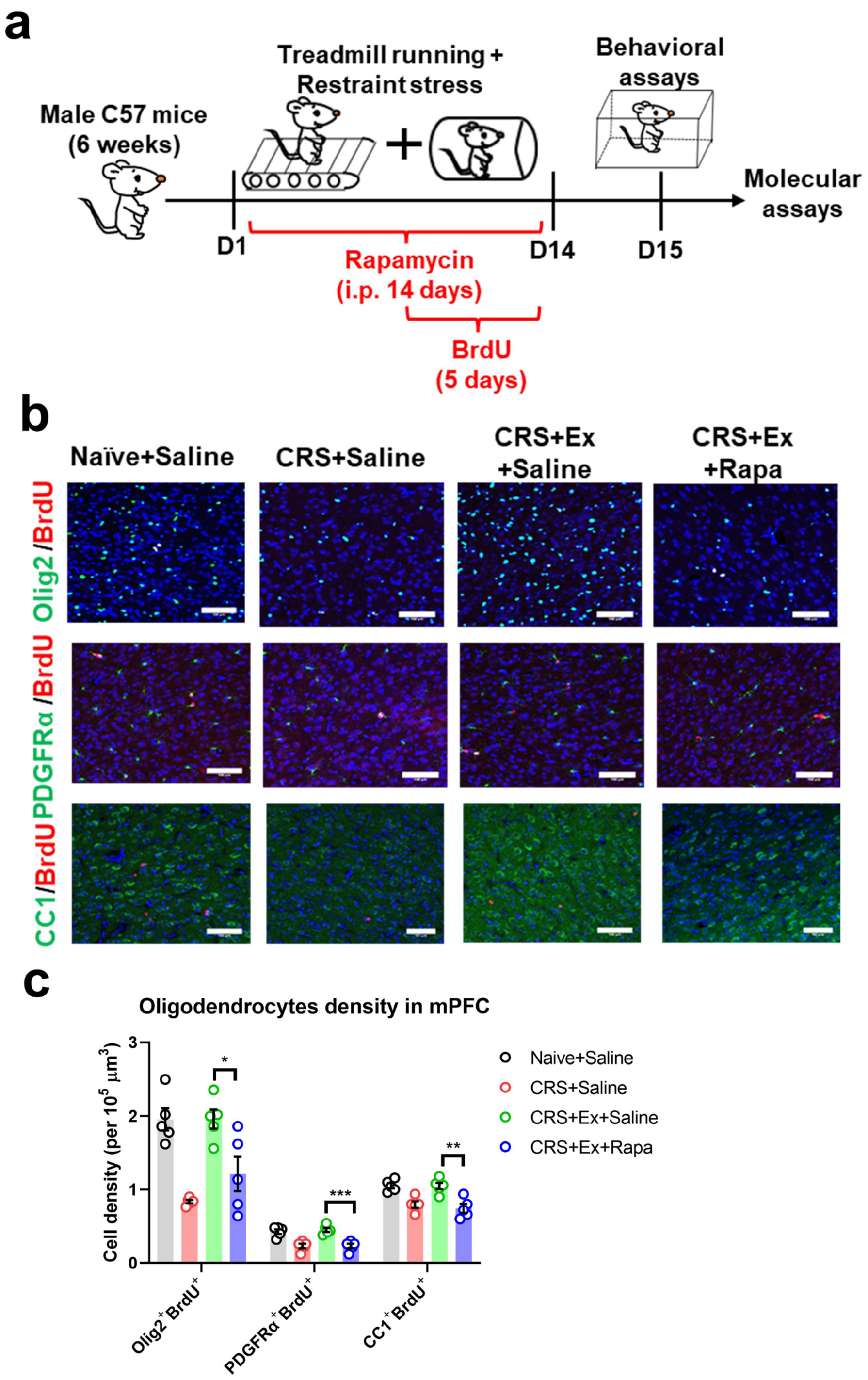

Fig. S3. Exercise activates mTOR pathway to direct oligodendrogenesis, related to Fig. 2.

(a) Illustrations for experimental designs.

(b) Fluorescent images showing the co-labelling of oligodendrocyte marker (Olig2, PDGFRα or CC1) with BrdU. Scale bar, 100 μm.

(c) The density of newly formed oligodendrocytes was repressed by rapamycin even in CRS+Ex group. Multiple *t*-test was used for comparisons between two groups. $^*P<0.05$, $^{**}P<0.01$, $^{***}P<0.001$. $N=5$ mice per group. All data were presented as mean±sem.

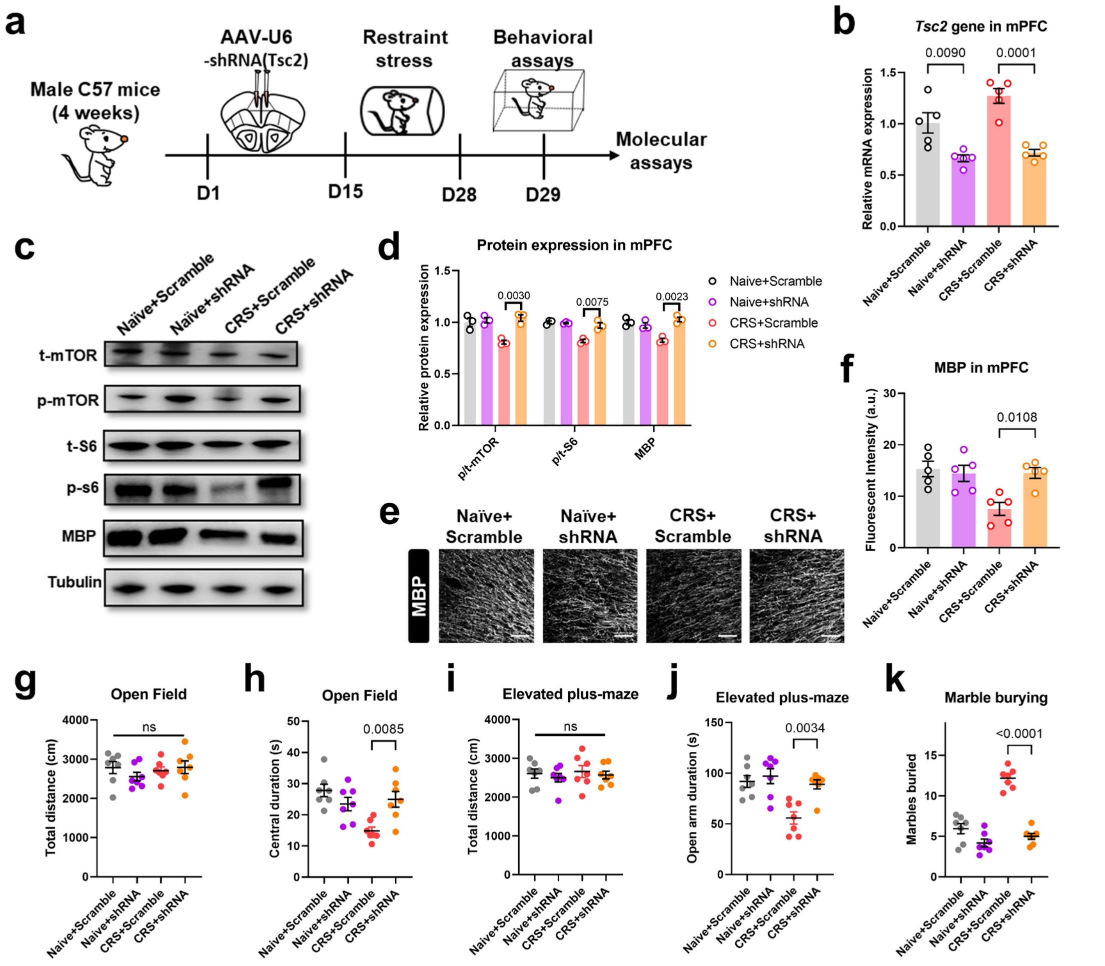

Fig. S4. Potentiation of brain mTOR pathway maintained axonal demyelination, related to Fig. 3.

(a) Schematic illustration for experimental designs of region-specific mTOR activation.

(b) Relative mRNA expression of *Tsc2* gene showed the effective knockdown by shRNA. One-way ANOVA, $F(3,16)=18.43$, $P<0.0001$. N=5 mice per group. Tukey's post-hoc test was used for comparisons between two groups.

(c) Representative Western blotting bands for mTOR proteins and MBP expression in PFC extracts.

(d) Quantification of relative protein expression showed the suppression of p-mTOR, p-S6 and MBP proteins when *Tsc2* gene was knocked down in mPFC. Multiple *t*-test was used for comparisons between two groups. N=3 mice per group.

(e) Fluorescent images of MBP in mPFC region showed the demyelination under *Tsc2* gene knockdown. Scale bar, 100 μm.

(f) Fluorescent intensity (in a.u.) of MBP was increased in CRS mice after *Tsc2* gene knockdown. One-way ANOVA, $F(3,16)=7.120$, $P=0.0030$. $N=5$ mice per group. Tukey's post-hoc test was used for comparisons between two groups.

(g) Total distance in the open field was unchanged under *Tsc2* gene knockdown. One-way ANOVA, $F(3,24)=0.680$, $P=0.5725$.

(h) Time spent in the central region in the open field was increased under *Tsc2* gene knockdown. One-way ANOVA, $F(3,24)=7.627$, $P=0.0009$.

(i) Total distance on the elevated plus maze was unchanged in CRS+shRNA group. One-way ANOVA, $F(3,24)=0.2921$, $P=0.8307$.

(j) Time duration in the open arm on the elevated plus maze was increased in CRS+shRNA group. One-way ANOVA, $F(3,24)=9.768$, $P=0.0002$.

(k) The number of marbles buried was decreased by suppressing *Tsc2* gene expression. One-way ANOVA, $F(3,24)=53.74$, $P<0.0001$.

$N=7$ mice in each group in **(g-k)**. Tukey's post-hoc test was used for comparisons between two groups.

All data were presented as mean±sem.

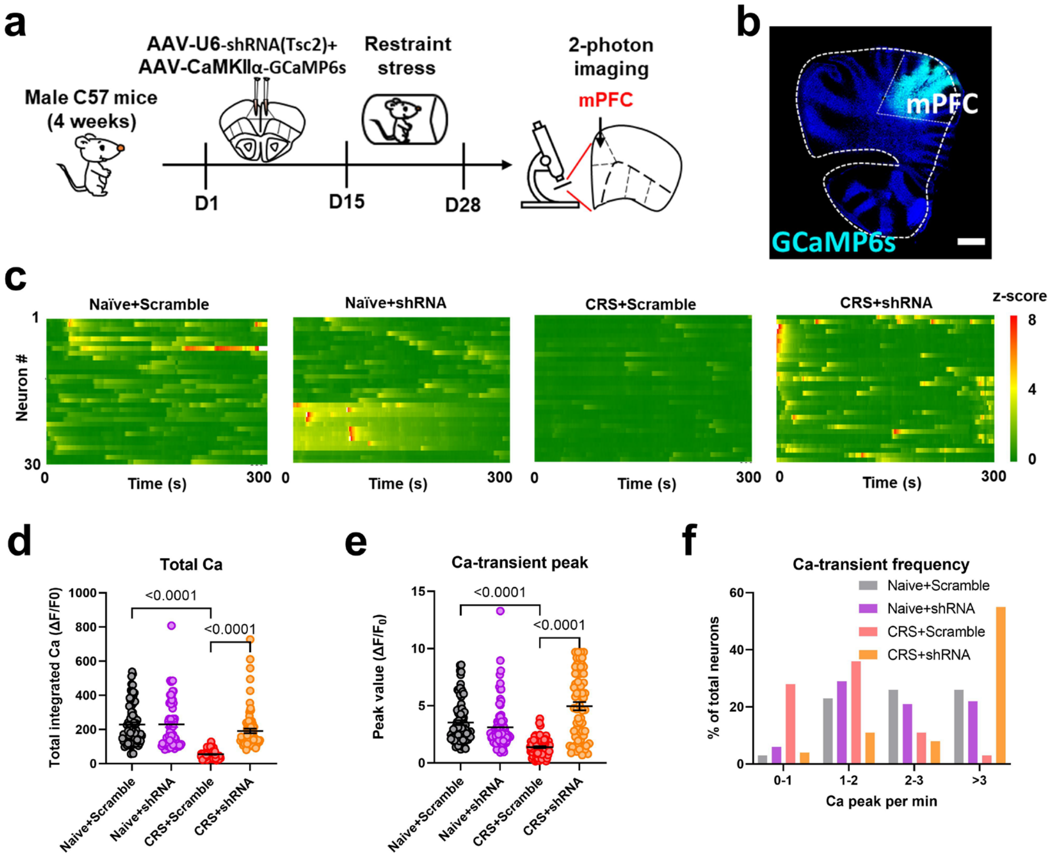

Fig. S5. Activating brain mTOR pathway potentiated neuronal activities, related to Fig. 4.

(a) Experimental flowcharts of *in vivo* 2-photon imaging assay.

(b) Expression of GCaMP6s in mPFC region. Scale bar, 500 μm.

(c) Heatmaps showing the *in vivo* calcium activities of mPFC neurons. A total of 30 neurons were displayed, with the calcium transients transformed as z-scores.

(d) Total integrated calcium levels of mPFC were decreased by CRS and re-elevated by *Tsc2* gene knockdown. Nonparametric Kruskal-Wallis test statistic=173.9, P<0.0001.

(e) Similar trends were observed for the peak values of calcium transients. Nonparametric Kruskal-Wallis test statistic=111.1, P<0.0001.

(f) Frequency distribution of calcium transient frequency (spike per min) also showed higher frequency by *Tsc2* gene knockdown.

n=80 neurons from 4 mice in each group in **(d-f)**. Dunn's test was used for the comparison between two groups.

All data were presented as mean±sem.

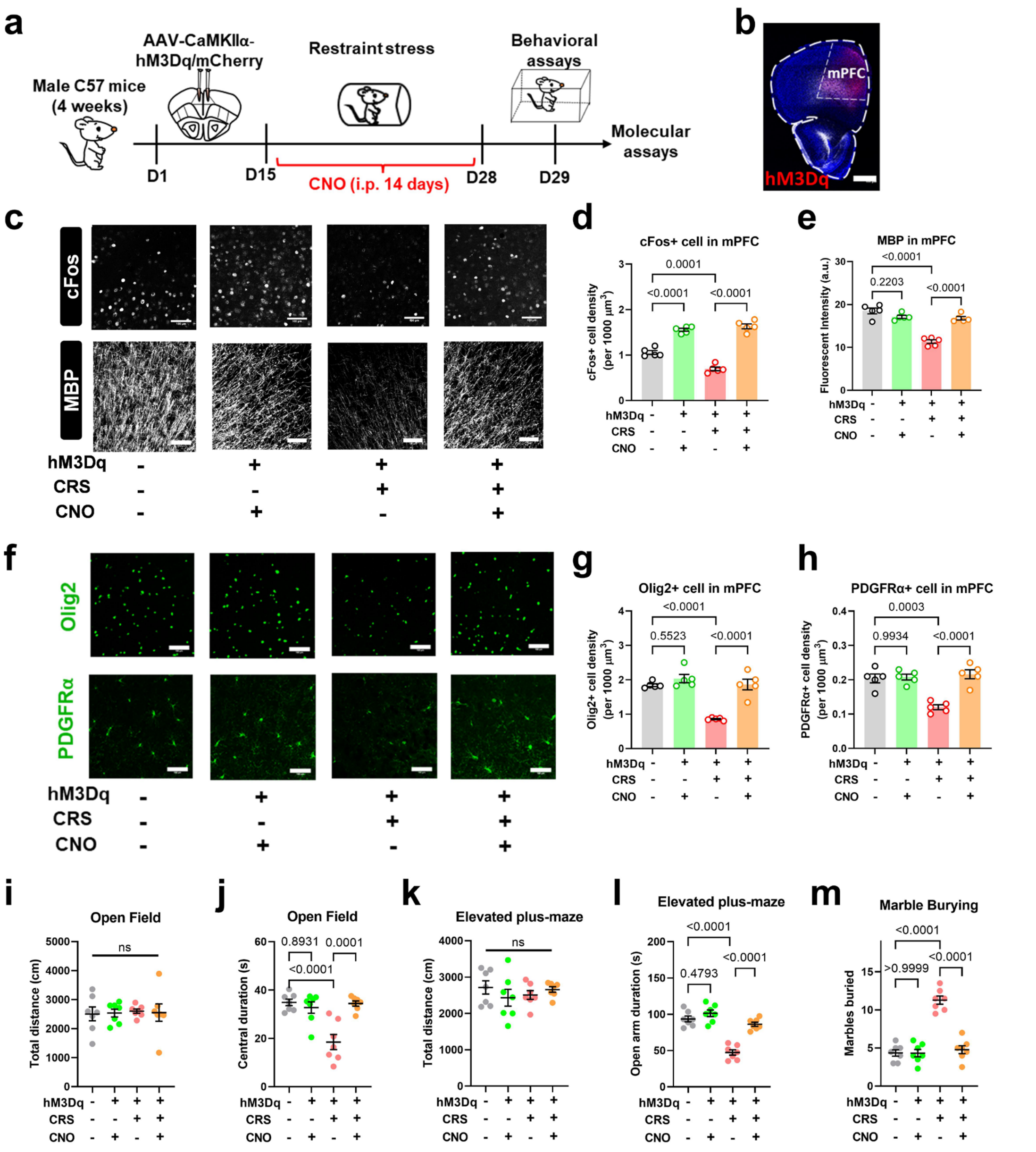

Fig. S6. Reactivating mPFC activity prevented demyelination and anxiety, related to Fig. 5.

(a) Experimental designs of chemogenetic activation assay.

(b) Transfection sites of AAV-hM3Dq into mPFC. Scale bar, 500 μm.

(c) Fluorescent images showing cFos (upper panels) and MBP (lower panels). Scale bar, 100 μm.

(d) Quantification of cFos suggested the inhibition of mPFC neurons under CRS, and the re-activation by CNO infusion. One-way ANOVA, $F(3,16)=107.5$, $P<0.0001$.

(e) MBP fluorescent intensity (in a.u.) was increased under neuronal activation. One-way ANOVA,

$F(3,16)=43.10$, $P<0.0001$.

(f) Fluorescent intensity of oligodendrocytes (Olig2 and PDGFRα) in mPFC. Scale bar, 100 μm.

(g) Quantification showed increased density of Olig2+ cells after reactivation of mPFC neurons. One-way ANOVA, $F(3,16)=28.02$, $P<0.0001$.

(h) The density of PDGFRα cells was increased in CNO treated animals. One-way ANOVA, $F(3,16)=17.34$, $P<0.0001$.

$N=5$ mice in each group in **(c-h)**. Tukey's post-hoc test was used for comparisons between two groups.

(i) Total distance in the open field was unchanged under mPFC neuron reactivation. One-way ANOVA, $F(3,24)=0.03745$, $P=0.9901$.

(j) Time spent in the central region in the open field was increased under mPFC neuron reactivation. One-way ANOVA, $F(3,24)=13.41$, $P<0.0001$.

(k) Locomotor activity on the elevated plus maze was unchanged in CRS+hM3D+CNO group. One-way ANOVA, $F(3,24)=0.6401$, $P=0.5966$.

(l) Time duration in the open arm on the elevated plus maze was increased in CRS+hM3D+CNO group. One-way ANOVA, $F(3,24)=39.93$, $P<0.0001$.

(m) The number of marbles buried was decreased by CNO treatment. One-way ANOVA, $F(3,24)=48.25$, $P<0.0001$.

$N=7$ mice in each group in **(i-m)**. Tukey's post-hoc test was used for comparisons between two groups.

All data were presented as mean±sem.

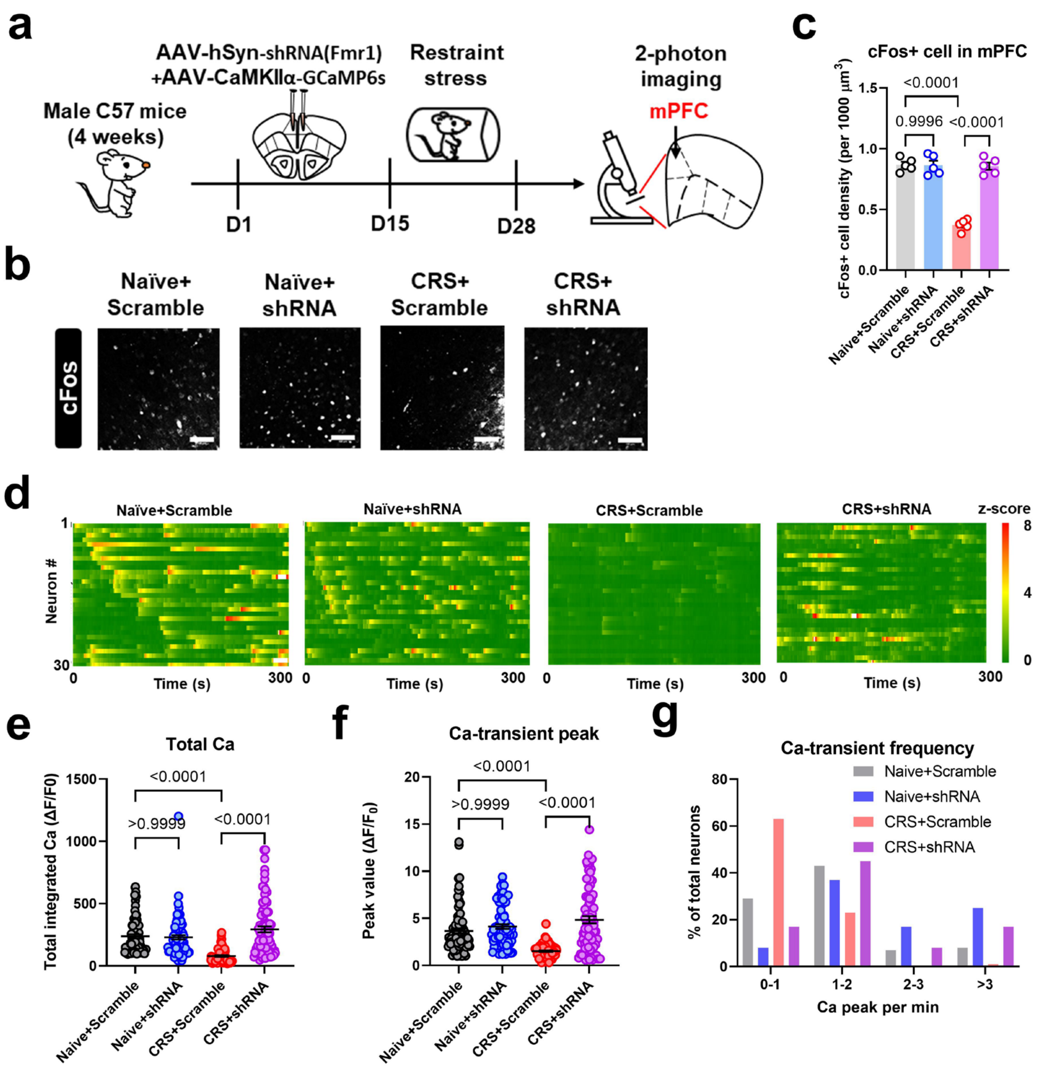

Fig. S7. Knocking down *Fmr1* gene potentiated mPFC neurons, related to Fig. 6.

(a) Experimental designs for the *in vivo* calcium imaging assays following *Fmr1* gene silence.

(b) Immunofluorescent images for cFos in mPFC. Scale bar, 100 μm.

(c) Quantification of cFos suggested the potentiation of mPFC neurons under *Fmr1* gene silence. One-way ANOVA, $F(3,16)=74.34$, $P<0.0001$. $N=5$ mice in each group. Tukey's post-hoc test was used for comparisons between two groups.

(d) Heatmaps showing the *in vivo* calcium activities of mPFC neurons. A total of 30 neurons were

displayed, with the calcium transients transformed as z-scores.

(e) Total integrated calcium levels of mPFC were re-elevated by inhibiting *Fmr1* gene expression. Nonparametric Kruskal-Wallis test statistic=116.9, $P<0.0001$.

(f) Similar trends were observed for the peak values of calcium transients. Nonparametric Kruskal-Wallis test statistic=99.42, $P<0.0001$.

(g) Frequency distribution of calcium transient frequency (spike per min) also showed elevated frequency by *Fmr1* gene knockdown.

n=80 neurons from 4 mice in each group in **(e-g)**. Dunn's test was used for the comparison between two groups.

All data were presented as mean±sem.

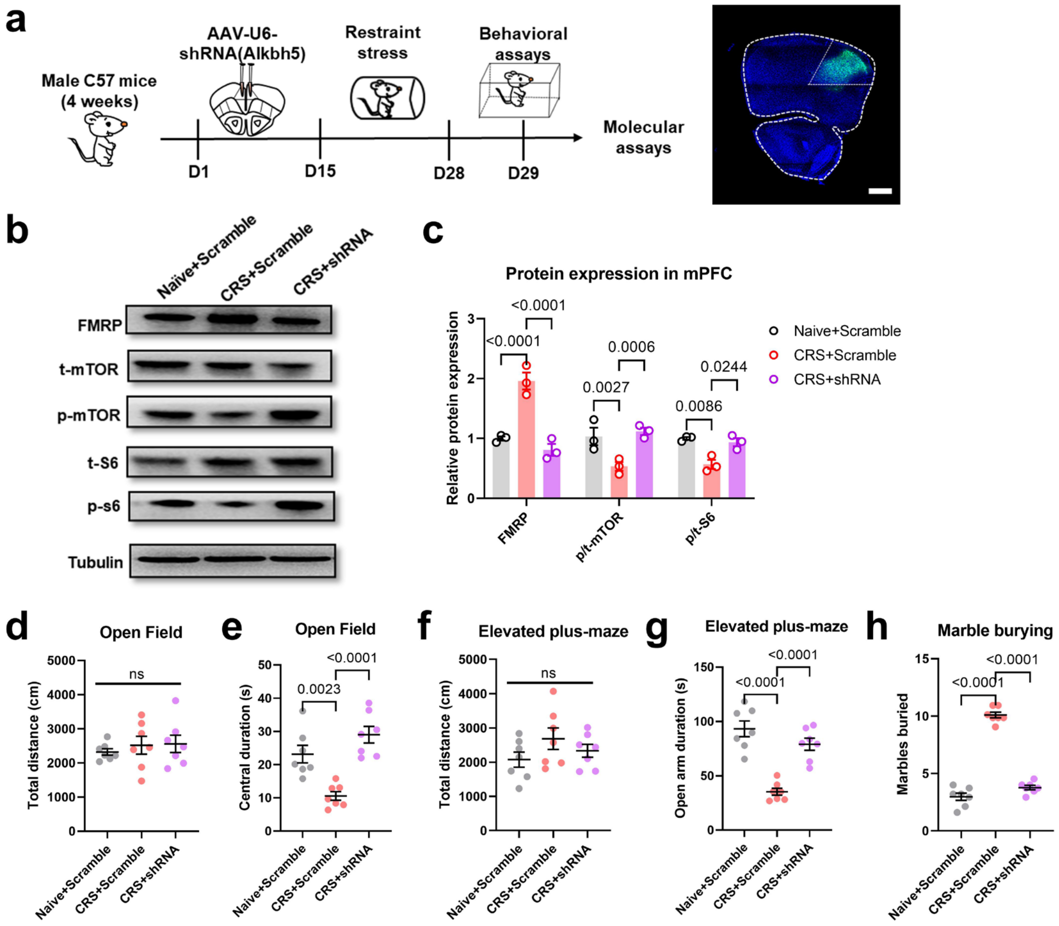

Fig. S8. Maintenance of brain RNA methylation exerted anxiolytic effects, related to Fig. 7.

(a) Experimental design for *Alkbh5* gene knockdown assay by locally transfecting shRNA. Right: Representative images of viral infection sites. Scale bar, 200 μm.

(b) Representative blotting bands for FMRP, p-mTOR and p-S6.

(c) Quantification analysis found that blocking ALKBH5 in CRS mice suppressed FMRP expression and activated mTOR pathway. Multiple *t*-test was used for comparisons between two groups. N=3 mice per group.

(d) Total distance travelled in the open field was unaffected. One-way ANOVA, $F(2,18)=0.3401$, $P=0.7162$.

(e) Time spent in the central region in the open field was increased under shRNA-*Alkbh5*. One-way

ANOVA, $F(2,18)=17.90$, $P<0.0001$.

(f) The general locomotor activity on the elevated plus maze was unchanged. One-way ANOVA, $F(2,18)=1.525$, $P=0.2444$.

(g) Time duration in the open arm on the elevated plus maze was increased in CRS+shRNA group. One-way ANOVA, $F(2,18)=29.14$, $P<0.0001$.

(h) The number of marbles buried was decreased by blocking *Alkbh5* gene expression. One-way ANOVA, $F(2,18)=227.6$, $P<0.0001$.

$N=7$ mice in each group in **(d-h)**. Tukey's post-hoc test was used for comparisons between two groups.

All data were presented as mean±sem.

Table S1. Antibodies, viral vectors and reagents used in all assays.

REAGENT	SOURCE	IDENTIFIER (CAT NO.)
**(i) Antibodies**		
anti-GAPDH	CST	5174s
anti-BDNF	ABCAM	ab108319
anti-FMRP	ABCAM	ab17722
anti-TUBULIN	CST	2146s
anti-mTOR	Cell Signaling Technology	#2972
anti-p-mTOR	Cell Signaling Technology	#2971
anti-p -TrkB	Thermo Fisher Scientific	PA5-36695
anti-TrkB	Thermo Fisher Scientific	#4603
anti-MBP	Thermo Fisher Scientific	#78896
anti-p-S6	Thermo Fisher Scientific	#4858
anti-S6	Thermo Fisher Scientific	#2217
anti-p-AKT	Thermo Fisher Scientific	#4060
anti-AKT	Thermo Fisher Scientific	#9272
anti-Olig2	Abcam	ab109186
anti-CC1	Calbiochem	OP80
anti-PDGFRalpha	R&D systems	AF1062
anti-Iba1	Wako	019-19741
anti-GFAP	Millpore	MAB360
anti-BrdU	Abcam	ab6326
anti-c-Fos	Cell Signaling Technology	Cat#2250; RRID: AB_2247211
Alexa Fluor 488 conjugated with Streptavidin-	Thermo Fisher Scientific	Cat#S32354; RRID: AB_2315383
Alexa Fluor 488 conjugated goat anti-rabbit IgG	Jackson ImmunoResearch	Cat#111-545-003; RRID: AB_2338046
DyLight 594 goat anti-mouse IgG	Thermo Fisher Scientific	Cat# 35510; RRID: AB_1185569
DyLight 647 goat anti-rabbit IgG	Vector Laboratories	Cat# DI-1649; RRID: AB_2336420
**(ii) Viral vectors**		**Serotype**
rAAV-U6-shRNA (Fmr1)-CMV-EGFP-SV40 pA	Brain VTA, Wuhan.	AAV2/8
rAAV-U6-shRNA (Scramble)-CMV-EGFP-SV40 pA	Brain VTA, Wuhan.	AAV2/8
rAAV -EF1a- P2A-mCherry-WPRE-hGH	Brain VTA, Wuhan.	AAV2/9
rAAV-U6-shRNA (Tsc2)-CMV-EGFP-SV40 pA	Brain VTA, Wuhan.	AAV2/9
rAAV-U6-shRNA (Raptor)-	Brain VTA, Wuhan.	AAV2/8

CMV-EGFP-SV40 pA		
rAAV-CaMKIIα-GCaMP6s-WPRE-hGH pA	Brain VTA, Wuhan.	AAV2/9
AAV- CaMKIIα -hM4D(Gi)-mCherry	Taitool BioScience Co, Shanghai	AAV2/9
AAV- CaMKIIα -hM3D(Gi)-mCherry	Taitool BioScience Co, Shanghai	AAV2/9
AAV- CaMKIIα -mCherry	Taitool BioScience Co, Shanghai	AAV2/9
**(iii) Chemicals, Peptides, and Recombinant Proteins**		
Clozapine N-oxide (CNO)	Sigma-Aldrich	Cat# C0832
K252a	Cell Signaling Technology	#12754
Rapamycin	abcam	ab141704
Doxycycline (DOX)	MCE	hy-n0565B
**(iv) Commercially purchased assay kits**		
PrimeScript™ RT Regeat Kit	Takara Bioscience	Cat# RR420A
SYBR Premix Ex Taq™	Takara Bioscience	Cat# RR037A
BCA Protein Assay Kit	Beyotime	p0012
EZ-10 TOAL RNA MINI-RPEPS	BBI	B618583
